# Pigment Epithelium-Derived Factor (PEDF) Fragments Prevent Mouse Cone Photoreceptor Cell Loss Induced by Focal Phototoxicity In Vivo

**DOI:** 10.3390/ijms21197242

**Published:** 2020-09-30

**Authors:** Francisco J. Valiente-Soriano, Johnny Di Pierdomenico, Diego García-Ayuso, Arturo Ortín-Martínez, Juan A. Miralles de Imperial-Ollero, Alejandro Gallego-Ortega, Manuel Jiménez-López, M. Paz Villegas-Pérez, S. Patricia Becerra, Manuel Vidal-Sanz

**Affiliations:** 1 Departamento de Oftalmología, Facultad de Medicina, Universidad de Murcia, e Instituto Murciano de Investigación Biosanitaria Virgen de la Arrixaca (IMIB-Arrixaca), 30.120 Murcia, Spain; fjvaliente@um.es (F.J.V.-S.); johnnydp@um.es (J.D.P.); diegogarcia@um.es (D.G.-A.); Arturo.OrtinMartinez@uhnresearch.ca (A.O.-M.); juanantoniomiralles@gmail.com (J.A.M.d.I.-O.); alejandrogallego@um.es (A.G.-O.); manu@um.es (M.J.-L.); mpville@um.es (M.P.V.-P.); 2 Donald K Johnson Eye Institute, Krembil Research Institute, University Health Network, Toronto, ON M5T 2S8, Canada; 3 Section of Protein Structure and Function, NEI-NIH, Bethesda, MD 20892-0608, USA

**Keywords:** PEDF, PEDF fragment 17-mer, PEDF fragment 17-mer[H105A], cone-photoreceptor, in vivo phototoxicity model, in vivo neuroprotection, adult mice, BDNF, bFGF

## Abstract

Here, we evaluated the effects of PEDF (pigment epithelium-derived factor) and PEDF peptides on cone-photoreceptor cell damage in a mouse model of focal LED-induced phototoxicity (LIP) in vivo. Swiss mice were dark-adapted overnight, anesthetized, and their left eyes were exposed to a blue LED placed over the cornea. Immediately after, intravitreal injection of PEDF, PEDF-peptide fragments 17-mer, 17-mer[H105A] or 17-mer[R99A] (all at 10 pmol) were administered into the left eye of each animal. BDNF (92 pmol) and bFGF (27 pmol) injections were positive controls, and vehicle negative control. After 7 days, LIP resulted in a consistent circular lesion located in the supratemporal quadrant and the number of S-cones were counted within an area centered on the lesion. Retinas treated with effectors had significantly greater S-cone numbers (PEDF (60%), 17-mer (56%), 17-mer [H105A] (57%), BDNF (64%) or bFGF (60%)) relative to their corresponding vehicle groups (≈42%). The 17-mer[R99A] with no PEDF receptor binding and no neurotrophic activity, PEDF combined with a molar excess of the PEDF receptor blocker P1 peptide, or with a PEDF-R enzymatic inhibitor had undetectable effects in S-cone survival. The findings demonstrated that the cone survival effects were mediated via interactions between the 17-mer region of the PEDF molecule and its PEDF-R receptor.

## 1. Introduction

Age-related macular degeneration causes progressive degeneration of the cones located in the macula and is the most common cause of irreversible blindness in the elderly [1,2]. The environmental risk factors in developing the disease are not yet fully established, but, in addition to smoking, obesity or hypertension, excessive exposure to sunlight is considered a determining factor in its development, precocity and severity, particularly at early stages [3]. Therefore, taking into account this risk factor, many animal models of light-induced retinal degeneration have been developed, and serve to study the evolution of the degeneration and to test possible protective strategies [4,5,6,7,8,9,10,11]. Several of these studies have documented the effect of excessive exposure to short-wave visible light (blue light) on the retina [11,12,13,14,15,16,17,18]. Blue light, the most damaging component of sunlight, causes oxidative stress that induces the loss of retinal pigment epithelium cells and photoreceptors [13,14,15,16,17,18]. We have recently characterized a new focal blue light emitting diode (LED) induced phototoxicity (LIP) model in albino mice that results in focal phototoxic cone degeneration in an area of the retina that coincides with the highest density of rodent photoreceptors [16,18,19,20]. This model allows automatic quantification of S-cone outer segments (OS) that is appropriate for determining the efficacy of different neuroprotective drugs [16,18].

One of the most promising approaches to reduce or slow down photoreceptor loss after phototoxicity is the use of neurotrophic factors, such as brain derived neurotrophic factor (BDNF), basic fibroblast growth factor (bFGF) or pigment epithelium-derived factor (PEDF) [16,18,21,22,23,24,25,26,27]. PEDF, a member of the superfamily of serine protease inhibitors (*SERPINs*) encoded by the *SERPINF1* gene, is involved in the differentiation, development, and survival of retinal neurons and in attenuating angiogenesis [16,23,26]. The sequence of the multifunctional protein has domains that confer neurotrophic and antiangiogenic activities to PEDF [28]. These domains are separate and independent regions, both of which are away from the *SERPIN* exposed loop involved in protease inhibition in other serpins, and interact with individual receptors for each activity [28]. Photoreceptors express the patatin-like phospholipase domain-containing 2 (*PNPLA2*) gene for PEDF-R, which is a neurotrophic receptor for PEDF [29,30]. PEDF-R is distributed in the inner segments of photoreceptors [21,29]. Small PEDF peptides from the neurotrophic domain bind PEDF-R and exhibit survival activities on retinal cells [21,23,31]. In this regard, the protective capacity of 17-mer (a peptide of 17 amino acids spanning positions 98–114 of the human PEDF sequence), and 17-mer[H105A] (a derived 17-mer peptide with single residue substitution to alanine on position 105 and improved retinoprotective activity) has been recently described in vivo and ex vivo in the *rd1*, *rd10* and *Mitf* mouse models of inherited genetic retinal degeneration [21,23,32]. A 17-mer peptide version with a single substitution at position 99 from arginine to alanine, 17-mer[R99A], does not have PEDF-R binding affinity nor its consequential neurotrophic activity. Although PEDF protects S-cones in a rat LIP model [16], and the cone-like 661 W cell line against light damage [33] and oxidative stress [34], it is not yet known whether these peptides have a protective effect on cones.

Here, we investigated the neuroprotective effects of intravitreal injections of PEDF-derived fragments on cones of mice induced to die by phototoxicity. The PEDF peptides were the 17-mer, 17-mer[R99A] and 17-mer[H105A]. Recombinant human PEDF, brain-derived neurotrophic factor and basic fibroblast growth factor were also used. We challenged the effectors with blocking peptide P1 derived from the PEDF-R that interferes with the interactions between PEDF and PEDF-R receptor and an enzymatic inhibitor of the receptor. We used modern techniques developed in our laboratory to identify and automatically count the population of S-cones [16,35] in a model of LIP cone-photoreceptor degeneration of mouse in vivo. We discuss the neuroprotective properties of a single intravitreal administration of PEDF-peptide fragments on LIP induced focal loss of cones in the albino mouse retina.

## 2. Results

### 2.1. Light Emitting Diode Induced-Phototoxicity (LIP) Model Results in the Loss of S-Cones in the Damaged Region

**Figure 1:**
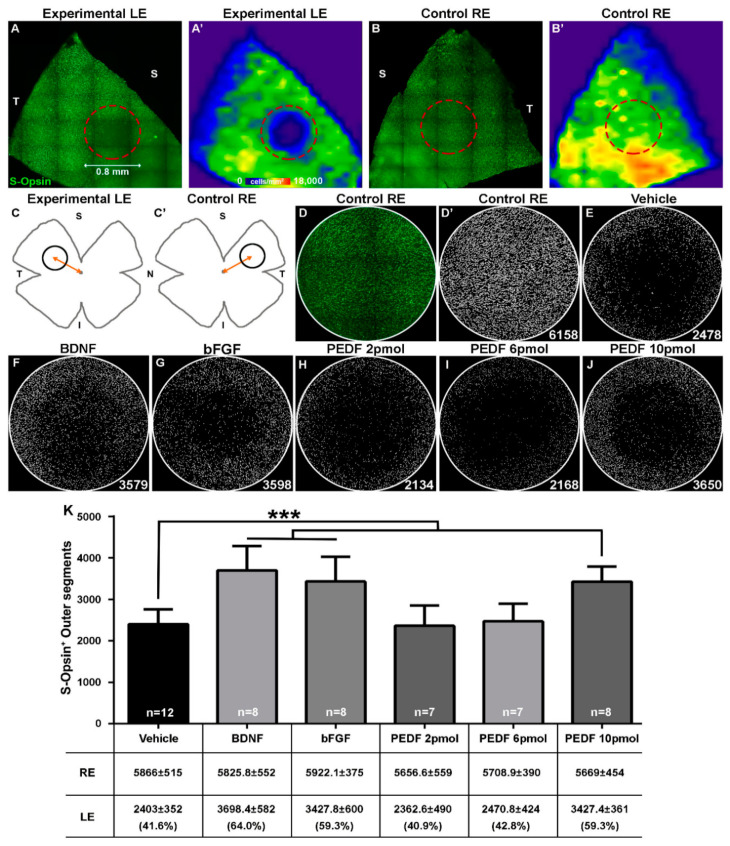
PEDF (pigment epithelium-derived factor) protection of S-cones against LIP (Light Emitting Diode-Induced Phototoxicity)-induced damage. Photomontages of supratemporal retinal quadrants and their corresponding isodensity maps in an experimental retina (left eye) exposed to LIP (**A**,**A’**) and its fellow-contralateral retina (right eye) (**B**,**B’**) immunostained with an antibody to detect S-opsin OS. The predetermined fixed-size circular area (PFA) involving the retinal damage resulting from LED exposure is outlined by a dotted red circle (diameter of 0.8 mm) on the left retina (**A**,**A’**) and its corresponding region on the right retina (**B**,**B’**). (**C**) and (**C’**) depict the location of the PFA for an experimental and its contralateral retina. Panel (**D**) shows OS stained with S-opsin of a right PFA. Panels (**D’–J**) show the S-opsin^+^OS automatically counted and represented as white dots in a representative right contralateral PFA (**D’**) and in representative experimental PFAs tested with vehicle (**E**), BDNF (**F**), bFGF (**G**) and PEDF at 2 pmol (**H**), 6 pmol (**I**) or 10 pmol (**J**). Panel (**K**) shows a bar graph and a table showing the S-opsin^+^OS that survive at 7 days after LIP in PFA treated with the indicated effectors (*x*-axis). The numbers of cones counted within the PFA are expressed as mean ± SD and in parentheses as the percentage of survival compared to the mean number of cones counted in all right contralateral PFAs of group A (5778 ± 454 S-opsin^+^OS; *n* = 50). (*** *p* < 0.001). S, superior. I, inferior. N, nasal. T, temporal.

Swiss mice were dark-adapted overnight, anesthetized, and their left eyes were exposed for 20 s (200 lux) to a blue (400 nm) LED placed over the cornea. The population of S-cones was then identified and counted automatically within the pre-determined fixed-size circular area (PFA) centered on the lesion in the left retinas and within corresponding regions of their fellow-right retinas. The LIP retinas (left eyes) showed a consistent circular area with a significant loss of S-cones within the supratemporal quadrant that was more pronounced in the center of the lesion (2403 ± 352 S-opsin^+^OS; *n* = 12 for Vehicle group) (Figure 1A,A’,E–J). The contralateral-fellow retinas (right eyes) that were not exposed to blue light showed the characteristic homogeneous distribution of S-opsin^+^OS throughout the retina with higher densities within the dorsal retina than the rest of the retina [16,18] (Figure 1B,B’,D,D’). Although these right retinas cannot be considered as controls due to the possible contralateral effects derived from the manipulation of the experimental eye, which has been widely demonstrated [36,37,38,39,40], the counts of S-opsin^+^OS within the right PFAs (5778 ± 454 S-opsin^+^OS; *n* = 50) were comparable among individuals of the different subgroups, and thus total counts were pooled and used for reference.

### 2.2. Intravitreal Administration of PEDF, BDNF or bFGF Decreases LED-Induced Cone Loss

Group A was designed to test the efficacy of human PEDF protein. The left eyes of each animal were exposed to LIP. Immediately after LIP, intravitreal injections of PEDF protein, as well as neurotrophic factors BDNF and bFGF as positive controls, were administered in the left eyes, while the right eyes did not receive injection nor blue light. After 7 days post-injection, the number of S-cones were determined in PFAs. Figure 1E–J shows a representative PFA for each group. Figure 1K shows the average number of cones within the PFA for PEDF, BDNF and bFGF of Group A (*n* = 50). PEDF at 10 pmol in LIP injured eyes increased the number of S-opsin^+^OS (3427 ± 361 S-opsin^+^OS, *n* = 8) relative to vehicle-injected eyes exposed to LIP (2403 ± 352 cells, *n* = 12) (*p* < 0.001). The numbers with lower doses of PEDF were similar to those values with vehicle (2 and 6 pmol; 2362 ± 490 S-opsin^+^OS, *n* = 7, and 2471 ± 424 S-opsin^+^OS, *n* = 7, respectively) (Figure 1). As expected, intravitreal administration of positive controls for LIP: BDNF (92 pmol) or bFGF (27 pmol) increased the number of S-cones (3699 ± 582 S-opsin^+^OS, *n* = 8, and 3428 ± 600 S-opsin^+^OS, *n* = 8, respectively) when compared to vehicle (*p* < 0.001) (Figure 1). Thus, intravitreal injections of human PEDF (10 pmol) promoted significant neuroprotection to mouse cones.

### 2.3. S-Cone-Protective Efficacy of PEDF Fragments

**Figure 2:**
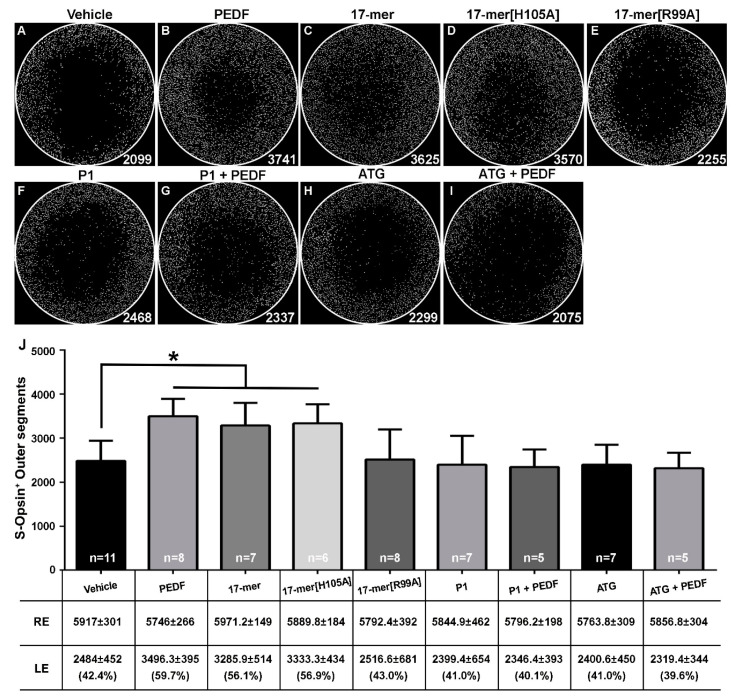
Effects of full-length PEDF and 17-mer, 17-mer[H105A] and 17-mer[R99A] peptides on LIP injured S-cones. Panels A-I show the S-opsin^+^OS automatically counted and represented as white dots in representative experimental PFAs tested with vehicle (**A**), full length PEDF (**B**), 17-mer (**C**), 17-mer[H105A] (**D**) and 17-mer[R99A] (**E**) peptides, P1 a blocking peptide, alone (**F**) or in combination with PEDF (**G**) or ATG, a PEDF-R inhibitor, alone (**H**) or in combination with PEDF (**I**). Panel (**J**) shows a bar graph and a table showing S-opsin^+^OS automatically counted and represented as white dots, that survive at 7 days after LIP in PFA treated with the different treatments indicated in the *x*-axis. The numbers of cones counted with PFA are expressed as mean ± SD and in parentheses as the percentage of survival compared to the mean number of cones counted all right contralateral PFAs of group B (5853 ± 310 S-opsin^+^OS; *n* = 64) (* *p* < 0.05).

Group B was designed to investigate the protective effects of fragments of PEDF in the LIP model. Figure 2 shows the representative PFAs for each effector of Group B. The average number of survived cones within the PFAs at 7 days post-injection and LIP are shown in Figure 2J (*n* = 64). We compared the number of S-cones in eyes treated with the peptides, and those with vehicles that survived LIP. Given that the full-length PEDF at 10 pmol was successful in the previous group, it was used as a positive neuroprotective control in this group (3496 ± 395 S-opsin^+^OS, *n* = 8; *p* < 0.01). The small PEDF-derived 17-mer fragment (10 pmol) was as effective in protecting S-cones (3286 ± 514 S-opsin^+^OS, *n* = 7; *p* < 0.05) as the full-length PEDF (10 pmol), both showing a higher survival of S-cones compared to vehicle (2484 ± 452 S-opsin^+^OS, *n* = 11). In addition, 17-mer[H105A] and 17-mer[R99A] peptides were tested. The protective effect was maintained with the 17-mer[H105A] injection (3333 ± 434 S-opsin^+^OS, *n* = 6) but not with the 17-mer[R99A], which resulted in comparable values to those obtained with vehicle (2517 ± 681 S-opsin^+^OS, *n* = 8), and was as expected for a peptide that does not have affinity for PEDF-R [31]. These observations imply that the 17-mer and 17-mer[H105A] peptides had efficacies similar to that of full-length PEDF, and that their cone-protective effect required interactions with PEDF-R.

To further investigate the requirement of PEDF-R on protection of S-cones damaged by LIP, we examined the effect of the PEDF-R blocker (P1) or the PEDF-R inhibitor atglistatin (ATG) on the PEDF-mediated S-cone protective activity. To block the PEDF:PEDF-R interactions, PEDF was preincubated with a molar excess of P1 peptide (molar ratio PEDF:P1 of 1:10) for 90 min at 4 °C before intravitreal injection. This combination resulted in decreasing the number of S-cones, similar to the results of vehicle (2346 ± 393 S-opsin^+^OS, *n* = 5), indicating that blocking the interaction with PEDF-R abolishes the PEDF activity on the cells. The S-cone numbers with injections of 100 pmol of P1 peptide alone were similar to those with vehicle (2399 ± 654 S-opsin^+^OS, *n* = 7), indicating that P1 by itself did not have an effect on cone numbers. This implies that the interaction of ligand to PEDF-R is required for the S-cone survival activity. We tested the effects of inhibiting the enzymatic activity of PEDF-R by injecting PEDF combined with ATG. ATG attenuated the survival activity of PEDF (PEDF + ATG, 2319 ± 344 S-opsin^+^OS, *n* = 5), and ATG alone resulted in similar values to those for vehicle (2401 ± 450 S-opsin^+^OS, *n* = 7). This implies that the enzymatic activity of PEDF-R was crucial for the PEDF-mediated S-cone survival activity. Altogether, the results demonstrated that the binding and enzyme activation of PEDF-R by the 17-mer region of PEDF was crucial for the protection of S-cones.

## 3. Discussion

This study describes that human PEDF prevents mouse cone photoreceptor cell loss induced by focal phototoxicity in vivo. The findings demonstrate that this activity is conferred by a neurotrophic region composed of amino acids that span between positions 98–114 of the human PEDF sequence. Furthermore, the 17-mer region interacts with the membrane-link PEDF-R to stimulate its enzymatic activity. The conclusion is derived from the similar efficacy of the small 17-mer, 17-mer[H105A] peptides and PEDF protein; the lack of activity of 17-mer[R99A]; loss of activity by interfering with the PEDF:PEDF-R interactions with P1 peptide; and by inhibiting the enzymatic activity of PEDF-R with ATG. This is the first report showing that PEDF peptides promote the survival of cones in vivo.

In the present study, retinas were analyzed at 7 days after LIP, at a time when retinal thinning, microglial response and photoreceptor apoptosis were already attenuated, and thus the neuroprotective effects of a given compound could be quantified [18]. In the first group (A) of mice, the dose of PEDF of 10 pmol attains neuroprotection which is similar to that of a higher dose of 92 pmol BDNF or 27 pmol bFGF, implying that PEDF has higher efficacy than the two well-known neuroprotective agents against LIP-induced cone degeneration [16,18,22,23,24]. Previous studies show that PEDF at 2, 6 and 10 pmol offers certain protection to photoreceptors undergoing death by a mutation of the *pde6* gene in *rd1* at 1 day post-injection, and the effects for longer periods of time are not available [31]. In another report, PEDF protected *rd10* photoreceptors for 3 days [23]. In the present study the effects remained for up to 7 days post injection, indicating a better protection by PEDF for cones dying due to phototoxicity.

Within the second group (B), we found that the small peptide 17-mer affords neuroprotection with equal efficacy as the full-length PEDF. Indeed, the neuroprotective effect of 10 pmol 17-mer is comparable to that of 10 pmol PEDF, which agrees with the idea that this short PEDF region contains necessary elements to promote cone-photoreceptor cell survival in vivo [23,31,32]. In addition, an effective protection was maintained with the 17-mer[H105A] peptide but lost with 17-mer[R99A], indicating that PEDF-R binding is required and arginine at position R99 is critical for S-cone protection. Finally, blocking the interactions between ligand and receptor for the PEDF/PEDF-R axis with P1 peptide and inhibiting PEDF-R with atglistatin conclusively demonstrated that the cone survival activity of PEDF is specifically mediated via the receptor PEDF-R in vivo. Thus, the interactions of the 17-mer region of PEDF are critical for the activation of PEDF-R for cone survival in the LIP model. These observations are consistent with the PEDF-mediated survival activities in rods of *rd1*, *rd10* and *Mitf* mice [21,23,31]. Supplementation of the active fragments of new synthetic peptides from one or several factors can be a potential treatment for the rescue and survival of photoreceptors. The rescue and survival of cones by PEDF administration suggests that this factor also contributes to recovery of cone function. This extrapolation is unknown and needs further study. Multiple electrode array (MEA) systems for recording ex vivo micro ERGs to assess focal retinal function in isolated mouse retinas, as described [41] may be instrumental to addressing the functional effects of PEDF on cone protection.

In summary, to the best of our knowledge, this is the first report documenting the neuroprotective effects of PEDF fragments in the blue LED focal photoreceptor injury mouse model in vivo. A single intravitreal injection of these PEDF molecules affords cone neuroprotection. Thus, these peptides may prove useful to further develop strategies to prevent cone-photoreceptor loss in inherited or acquired retinal degenerations.

## 4. Materials and Methods

### 4.1. Animal Handling

To carry out these experiments, we used 114 adult female albino mice (Swiss strain, 25–30 g) obtained from Charles River Laboratories (Barcelona, Spain). They were housed in the animal facilities of the University of Murcia (UM) in temperature- and light-controlled rooms (12 h light/dark cycle) with food and water “ad libitum”. Animal care and experimental procedures followed the ARVO and European Union guidelines for the use of animals in research and were approved by the Ethical and Animal Studies Committee of the UM (number: A13170110 and A13170111 approved in January 11, 2017). A mixture of xylazine (10 mg/kg body weight; Rompun^®^; Bayer, Kiel, Germany) and ketamine (60 mg/kg body weight; Ketolar^®^; Pfizer, Alcobendas, Madrid, Spain) was intraperitoneally administered for an anaesthetic use. After the eye manipulations, the eye was covered with an ointment to avoid drying of the cornea (Tobrex; Alcon S. A., Barcelona, Spain). For euthanasia, an overdose of pentobarbital (Dolethal, Vetoquinol^®^, Especialidades Veterinarias, S.A., Alcobendas, Madrid, Spain) was administered to previously anesthetized mice.

### 4.2. Light Emitting Diode-Induced Phototoxicity Model

The blue LIP model was performed as described before [18]. In brief, mice were dark-adapted overnight and one hour prior to LIP, the left eye was dilated using Tropicamida 1%^®^ (Alcon-Cusí, S.A., El Masnou, Barcelona, Spain). A blue LED (emission spectrum 390–410 nm; catalogue number 454–4405; Kingbright Elec. Co., Taipei, Taiwan) was placed 1 mm from the apex of the left cornea, with the necessary inclination to focus on the supratemporal region of the retina, and the mouse’s head was secured in a head-holder. The blue LED, manipulated from a computer and controlled with a luxometer (light meter TES-1330; TES Electrical Electronic Corp., Taipei, Taiwan) was presented for 20 s at 200 lux. LIP was performed between 10 am–12 am to homogenize conditions and control possible alterations by circadian rhythms [42,43]. In this work, the left retinas were used as experimental samples, with the contralateral-right ones kept intact.

### 4.3. Administration of the Neuroprotective Effectors

Immediately after LIP, the left eye received an intravitreal injection of 2.5 μL of different compounds per eye as follows. A first group (A) was designed to test the efficacy of PEDF protein. A total of 2, 6 or 10 pmol of recombinant human PEDF (*n* = 7–8; MW 50-kDa; purified as described [44]) diluted in phosphate buffered saline (PBS) was injected. As positive controls for neuroprotection and comparison, 92 pmol of recombinant human BDNF in PBS (*n* = 8; MW 27-kDa; 450-02, Peprotech^®^, London, UK), or 27 pmol of recombinant human bFGF (*n* = 8; MW 18-kDa; 100-18B, Peprotech^®^, London, UK) diluted in 2 mM Tris-Cl pH 7.6 were administered. A second group (B) was designed to test the efficacy of the small peptides 17-mer from the PEDF neurotrophic region (10 pmol; *n* = 7), altered 17-mer[R99A] (10 pmol; *n* = 8), and 17-mer[H105A] (10 pmol; *n* = 6), chemically synthesized as previously described [31]. Stocks of these compounds were diluted in PBS before intravitreal injection. Full length recombinant human PEDF (10 pmol; *n* = 8) was used as positive control. To challenge the PEDF neuroprotective effects, a synthetic peptide P1 derived from PEDF-R [27] and an enzymatic inhibitor of PEDF-R, atglistatin (ATG) [31] were tested. Peptide P1 was composed of residues Thr(210)-Leu(249) of human PEDF-R [27], and ATG was purchased from Sigma-Aldrich Inc^®^ (Quimica SL, Madrid, Spain). Peptide P1 was mixed in 10-fold molar-excess to the factor and pre-incubated for 30 min before injection to ensure complex formation. We administered synthetic peptide P1 (100 pmol; *n* = 7) or ATG (100 pmol; *n* = 7), alone or in combination with 10 pmol PEDF (P1 + PEDF; *n* = 5) or (ATG + PEDF; *n* = 5). Control mice were treated intravitreally with 2.5 μL of the corresponding vehicle solution for vehicle controls. The results of the vehicles were pooled, and the averages presented unified since there were no differences among them (Group A, *n* = 12; Group B, *n* = 11). Additionally, the fellow-right eyes were used as reference and their results unified within each group, A (*n* = 50) and B (*n* = 64), respectively.

### 4.4. Tissue Processing and Retinal Analysis

Seven days after LIP, mice were perfused transcardially and briefly with saline, followed by 4% paraformaldehyde in 0.1 M pH 7.2 phosphate buffer, and then retinal whole-mounts were prepared with the vitreous-side down, following a standard protocol previously developed in our laboratory [5,18,45,46]. Retinal wholemounts were used to immunodetect S-opsin with a primary antibody against S-opsin (goat anti-OPN1SW; 1:1000; Santa Cruz Biotechnologies), which in the albino mouse retina recognize ≈96% of all cone outer segments [18,20,35]. This was followed with a secondary antibody Alexa Fluor-488 donkey anti-goat (1:500; IgG (H + L), Molecular Probes, Invitrogen). Then, the images of immunostained retinas were acquired with a fluorescence microscope (Axioscop 2 Plus; Zeiss) following standard protocols previously developed in our laboratory [35]. LIP retinas consistently exhibited a circular region without S-opsin staining corresponding to a lesion lacking cones within the supratemporal quadrant, whose center was located ≈1.2 mm from the optic disc as shown in Figure 1A,A’. The surviving S-cones (S-opsin^+^OS) were automatically counted within a PFA of a 0.4 mm radius, centered on the lesion (Figure 1A,A’,C) following standard methods developed in our laboratory [18]. In the fellow-right retinas, the S-opsin^+^OS were counted with the same protocol in the corresponding supratemporal region for each mouse (Figure 1B,B’,C’).

### 4.5. Statistical Analysis

Data are presented as mean ± standard deviation (SD) and differences are considered significant when *p* < 0.05 (ANOVA, post hoc Tukey-test, GraphPad Prism v.7 (GraphPad, San Diego, CA, USA)).

## Abbreviations

LIP	Light emitting diode-induced phototoxicity
OS	Outer Segments
PEDF	Pigment epithelium-derived factor
BDNF	Brain derived neurotrophic factor
bFGF	Basic fibroblast growth factor
P1	PEDF-R blocker
ATG	Atglistatin
PFA	Pre-determined fixed-size circular area
